# Control Protocols for Range-Based Navigation of a Networked Group of Underwater Vehicles

**DOI:** 10.3389/frobt.2020.519985

**Published:** 2020-12-09

**Authors:** Daniela De Palma, Giovanni Indiveri, Gianfranco Parlangeli

**Affiliations:** ^1^Department of Innovation Engineering (DII), University of Salento (Interuniversity Center of Integrated Systems for the Marine Environment node), Lecce, Italy; ^2^Department of Informatics, Bioengineering, Robotics, and Systems Engineering (DIBRIS), University of Genova (Interuniversity Center of Integrated Systems for the Marine Environment node), Genova, Italy

**Keywords:** autonomous underwater vehicles, multi-agent system, relative localization, active estimation, range-based navigation

## Abstract

This paper tackles the problem of formation reconstruction for a team of vehicles based on the knowledge of the range between agents of a subset of the participants. One main peculiarity of the proposed approach is that the relative velocity between agents, which is a fundamental data to solve the problem, is not assumed to be known in advance neither directly communicated. For the purpose of estimating this quantity, a collaborative control protocol is designed in order to mount the velocity data in the motion of each vehicle as a parameter through a dedicated control protocol, so that it can be inferred from the motion of the neighbor agents. Moreover, some suitable geometrical constraints related to the agents' relative positions are built and explicitly taken into account in the estimation framework providing a more accurate estimate. The issue of the presence of delays in the transmitted signals is also studied and two possible solutions are provided explaining how it is possible to get a reasonable range data exchange to get the solution both in a centralized fashion and in a decentralized one. Numerical examples are presented corroborating the validity of the proposed approach.

## 1. Introduction

Localization is one of the most important basic abilities for an autonomous vehicle to perform autonomously a wide number of tasks (Ferri et al., 2017; Simetti et al., 2017; Antonelli et al., 2018), so that an accurate and reliable localization algorithm is a key practical tool for the success of mission in many applications of underwater robotics.

In essence, the localization problem is often addressed exploiting geometrical relations between the pose of the vehicle and the sensors, so that the issue of solving the localization problem may be strongly related to the environment of the given application. Sensor technology strongly depends on the environment, e.g., the Global Navigation Satellite System (GNSS), Attitude Heading Reference Systems (AHRS), radar-based tracking systems, accelerometers, gyros, and compass devices. This makes the issue of the underwater localization problem more challenging, and it has been considerably studied in the past years. Underwater acoustic-based trilateration solutions as long base line (LBL) systems have been studied as well, but they require complex deployment operations (Scherbatyuk, 1995).

Localization is a long-time debated research area in robotics and beyond, and different aspects have been studied over time. In this paper, we consider the relative localization problem for a team of agents, that is, the formation reconstruction problem in a multi-vehicle framework. This is a peculiar problem in the research area of localization, which has been recently considered by several authors for its importance in various applications. In the paper by Soares et al. (2013), the authors propose a formation keeping under severe communication and localization constraints, which is a typical condition of the underwater environment. In Sarras et al. (2017), the authors adopt an observer-based approach to treat the problem of multi-vehicle collaborative localization using time-varying range and relative velocity measurements, while in Halsted and Schwager (2017) a method of estimating the shape of an indoor environment using the echos of acoustic pulses among the robots is studied. Indeed, when an underwater mission is performed by a team of robots, it is often fundamental to know the relative positions and orientations in order to be able to correctly merge the data of the environment (e.g., merging pieces of map) collected by each individual robot (see, e.g., Roumeliotis and Bekey, 2000).

From a theoretic standpoint, the range-based estimation problems have been recently considered also in a single vehicle framework (Bayat et al., 2016). The challenges of dealing with single range measurements come from the fact that they are a non-linear algebraic map of the vehicles' positions hence the observability analysis requires the tools of local and weakly local observability (Hermann and Krener, 1977), but this approach suffers from several difficulties (Gadre and Stilwell, 2004; Ross and Jouffroy, 2005; Jouffroy and Reger, 2006). However, an alternative approach has been recently investigated recurring to a reformulation of the problem, which requires the observability of a linear time varying system (see, e.g., Batista et al., 2011; De Palma et al., 2017) so that a number of useless drawbacks of the local approach are avoided.

In this paper, we afford the relative positions reconstruction problem for a team of collaborative robots using local data. Collaborative navigation based on single-range data has been studied in the underwater environment (Fallon et al., 2010; Soares et al., 2013; Webster et al., 2013) as well as in more general settings (Cao et al., 2011). Indeed, since the milestone paper by Sanderson (1997), the area of cooperative navigation and localization has been significantly explored. One first significant attempt to the decentralized collective localization problem is explained in Roumeliotis and Bekey (2002); to achieve this goal, data processed during each collective localization session are propagated among all the robots in the group. This approach is further investigated in Mourikis and Roumeliotis (2006), where the Relative Position Measurement Graph (RPMG), i.e., the weighted directed graph representing the network of robot-to-robot exteroceptive measurements, is introduced and used as a key tool for the analysis of cooperative localization. The distributed acoustic navigation problem for Autonomous Underwater Vehicles (AUVs) is explored in Bahr et al. (2009), where the authors use acoustic ranging and data exchange based on dead-reckoning and range-only measurements provided by acoustic modems that are mounted on each vehicle to achieve cooperative positioning. In the paper by Allotta et al. (2014), the use of AUVs with low-cost instrumentation is explored (namely, each of them is equipped with a low-cost IMU, a compass and depth sensor, but only one of them, the master, has a high accuracy navigation sensor such as the DVL), and acoustic modems for communication are used as sensors of relative distance to achieve an innovative cooperative localization algorithm. In Soares et al. (2017), the authors optimize the non-convex maximum-likelihood estimator in the presence of range measurements contaminated with Gaussian noise, and obtain a convergent, accurate, and distributed positioning algorithm that outperforms the extended Kalman filter. However, this topic has been largely explored by several authors, and the interested reader may refer to Arai et al. (2002) (section V).

The research activity reported in this paper stems from the above considerations and is strongly inspired by the experience gained within a European project (Antonelli et al., 2018). The goal is to extend the preliminary results achieved by the same authors in De Palma et al. (2015) and De Palma et al. (2019) as follows. One of the main novelties with respect to De Palma et al. (2015) is relative to the following fact. Based on the consideration that communications networks in the underwater environment do not perform well, we want to avoid the direct communication between vehicles of the relative velocity by setting a suitable agreed control protocol in which it is possible to encapsulate the data which one vehicle wishes to communicate as a parameter that can be easily estimated using the relative motion by any of its neighbors. As opposed to the approach in Mourikis and Roumeliotis (2006), in this paper the solution proposed relies on intra-vehicle ranges only rather than relative positions of vehicles. As a further peculiar feature of the approach proposed by the authors of this paper, we further use topology-based relations among the unknown variables as an additional constraint and this results in reduction of the overall estimate uncertainty. Further, in this paper we explicitly account for delays in range measurements acquisition. Indeed, the technology underneath underwater networks is typically acoustic and communication delays may be significant and their impact may not be neglected. The solution provided in this paper exploits the intuitive idea of a neat time-division protocol to prevent any delay-related issue in the localization solution provided that an upper bound is available.

The paper is structured as follows: after a brief summary of notation and terminology in section 2, we provide the general problem statement in section 3, where section 4 is dedicated to the localization-oriented control protocol. In section 5, the observer design is performed, and section 5.1 is fully dedicated to the projection approach, which allows to improve the estimate precision. In section 6, the issue of delays in measurements is faced, and in section 7, two possible communication protocols are provided both in the case of a single “leader” agent performing the elaboration (thus only one agent collecting all the estimates) or any agent of the network. In section 8, a wide simulation activity is reported and discussed, showing the effectiveness of the proposed approach. Section 9 closes the paper summarizing the results achieved in the paper.

## 2. Notation and Graph Theory Terminology

In the following, we introduce the notation adopted in the paper and some tools from graph theory (Godsil and Royle, 2001), which are useful for a mathematical treatment of the problem. We use the symbol ⊗ to denote the Kronecker product between two matrices, which is defined as follows. For a pair of matrices ***A*** ∈ ℝ^*n*×*m*^ and ***B*** ∈ ℝ^*p*×*q*^:

(1) $$\begin{array}{l} A⊗B=\left[\begin{array}{ccc} a_{11}B & \cdots & a_{1m}B\\ \vdots & \ddots & \vdots \\ a_{n1}B & \cdots & a_{nm}B \end{array}\right]. \end{array}$$

We use diag(***A***_1_, …, ***A***_*n*_) to denote a block-diagonal matrix with matrix diagonal entries ***A***_*i*_. A *graph* G is the collection of a set V={1,…,n} called *set of nodes* and another set E⊆V×V, which is called *the set of edges*. For a given i∈V, the set $N_{i}=\left\{j\in V:\left(j,i\right)\in E\right\}$ is called the set of its *neighbors*. A path P between node *i* and node *j* is a collection of nodes and edges of G connecting *i* and *j*; a graph G=(V,E) is connected if there exists a path connecting each h,g∈V. A cycle is analogously defined with the additional condition that *i* = *j*. A cycle $\bar{C}$ is linearly independent from a preassigned set of cycles if at least one edge in $\bar{C}$ is not present in the union of the edge sets of the cycles.

## 3. Problem Formulation

Let ${\text{x}}_{i}\in {\mathbb{R}}^{3}$ for *i* = 1, 2, …, *n* denote the position of *n* agents and ${\text{v}}_{i}\in {\mathbb{R}}^{3}$ their velocity. Each agent is able to know its own velocity with reference to the common frame I so that, unless specified, we assume that the velocity is expressed in this common frame I. We assume that if two agents are able to measure the range between themselves, they are connected, so that it is possible to define a connection graph. Inspired by the work of Mourikis and Roumeliotis (2006), we refer to it as to an RPMG, which we assume to be a simple graph G with node set V={1,…,n} and the edges set E. We further assume that if two agents are able to measure the range between themselves, they can establish a communication link to exchange data, so that it is possible to consider G also a communication graph.

The evolution of the agents can be computed using simple kinematic equations:

(2) $$\begin{array}{l} {\overset{∙}{\text{x}}}_{i}\left(t\right)={\text{v}}_{i}\left(t\right)\;:\;i\in V \end{array}$$

(3) $$\begin{array}{l} {\text{z}}_{ij}\left(t\right)\;:\;={\text{x}}_{i}\left(t\right)-{\text{x}}_{j}\left(t\right)\;:\;\left(i,j\right)\in E \end{array}$$

(4) $$\begin{array}{l} {\text{v}}_{ij}\left(t\right)\;:\;={\text{v}}_{i}\left(t\right)-{\text{v}}_{j}\left(t\right)\;:\;\left(i,j\right)\in E \end{array}$$

so that

(5) $$\begin{array}{l} {\overset{∙}{\text{z}}}_{ij}\left(t\right)={\text{v}}_{ij}\left(t\right)\;:\;\left(i,j\right)\in E \end{array}$$

(6) $$\begin{array}{l} y_{ij}\left(t\right)=\|{\text{z}}_{ij}\left(t\right){\|}^{2}\;:\;\left(i,j\right)\in E, \end{array}$$

where **z**_*ij*_ in Equation (3) denotes the relative positions among those agents able to exchange information. All agents are assumed to be able to acquire measurements of their relative Euclidean distance *y*_*ij*_ in Equation (6), with the goal of estimating **z**_*ij*_ performing an elaboration of the relative range measurements *y*_*ij*_ and local data. A fundamental difference between this problem statement and the one afforded in the paper by De Palma et al. (2015) is that here we do not assume to exchange the velocity data through a dedicated underwater network, but we encapsulate this information as parameters of an agreed control protocol and infer the velocity value using a range-based Kalman filter as detailed in the following. We consider this choice of the problem statement a significant step forward for all those applications where only range information exchange is possible.

From now on we work under the assumption that the communication graph is time invariant. This choice is instrumental to keep the problem simple and the associated solution clear. The authors believe that it is a mild assumption considering that the resulting localization procedure requires a bounded amount of time. It is equivalent to assume that nodes that are neighbors at the initial time $\bar{t}$ keep this communication alive during the whole time span, while other nodes that may fall in the communication range after $\bar{t}$ are not included in the elaboration. Furthermore, from a practical point of view, it should be emphasized that acoustic underwater communications degrades drastically after certain threshold distances. If a group of underwater vehicles keeps its formation during a mission within such a threshold distance (most common case), the quality of the communications can be assumed to remain good and the communication links can be considered constant. Finally, even if a communication link (i,j) was lost, the proposed strategy could still be adopted by deleting the corresponding state variable **z**_*ij*_.

**Figure 1:**
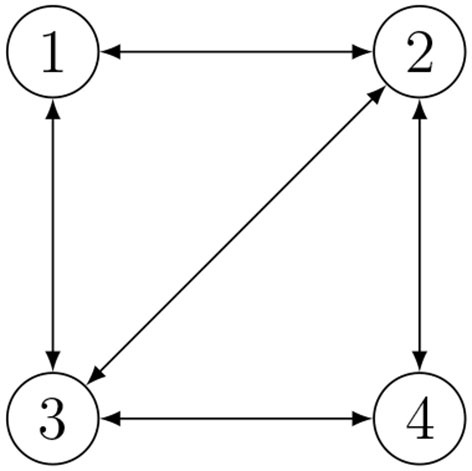
Example of Relative Position Measurement Graph (RPMG) with 4 agents and 5 links.

We now describe a strategy to improve the estimation when cycles are present in the communication graph. Indeed, the relative positions may be not independent, but they can be subject to geometric constraints if they belong to the same cycle. Based on the consideration that the sum of all the vectors representing the relative positions of agents belonging to a cycle must necessarily be zero, each set of independent cycles corresponds to a set of independent geometric constraints on the relative positions. Considering a connected graph with *n* nodes and *m* edges, any cycle basis can be mapped into a set of *m*−*n*+1 additional relations. A team of *n* = 4 agents with *m* = 5 links is depicted in Figure 1. It is possible to set *m*−*n*+1 = 2 additional relations, namely

(7) $$\begin{array}{l} {\text{z}}_{12}+{\text{z}}_{23}+{\text{z}}_{31}=0_{3\times 1} \end{array}$$

(8) $$\begin{array}{l} -{\text{z}}_{23}+{\text{z}}_{24}+{\text{z}}_{43}=0_{3\times 1} \end{array}$$

that can be rewritten as $D{\text{z}}^{*}=0_{3\times 1}$ with

(9) $$\begin{array}{l} D=\left[\begin{array}{ccccc} I_{3\times 3} & 0_{3\times 3} & 0_{3\times 3} & I_{3\times 3} & 0_{3\times 3}\\ 0_{3\times 3} & I_{3\times 3} & I_{3\times 3} & 0_{3\times 3} & -I_{3\times 3} \end{array}\right]\in {\mathbb{R}}^{6\times 15} \end{array}$$

(10) $$\begin{array}{l} z^{*}={\left(z_{12}^{⊤}\;z_{24}^{⊤}\;z_{43}^{⊤}\;z_{31}^{⊤}\;z_{23}^{⊤}\right)}^{⊤}\in {\mathbb{R}}^{15} \end{array}$$

The above example can be easily generalized, and it is possible to write a general setting as follows. For a team of *n* nodes and RPMG edge set E, the additional relations can be expressed as:

(11) $$\begin{array}{l} Dz^{*}=0_{3\left(m-n+1\right)\times 1} \end{array}$$

being

(12) $$\begin{array}{l} {\text{z}}^{*}={\left(\cdots {\text{z}}_{ij}^{⊤}\cdots \;\right)}^{⊤}\in {\mathbb{R}}^{3m}\text{with}\left(i,j\right)\in E, \end{array}$$

(13) $$\begin{array}{l} D=A⊗I_{3\times 3}, \end{array}$$

(14) $$\begin{array}{l} A=\left(a_{lk}\right)\;\left(l=1,\ldots ,m-n+1;k=1,\ldots ,m\right) \end{array}$$

where ***D*** ∈ ℝ^3(*m*−*n*+1) ×3*m*^, ***A*** ∈ ℝ^(*m*−*n*+1) × *m*^ and ***A*** is a signed structured (0, 1) matrix, namely *a*_*lk*_ ∈ {−1, 0, +1}. Each geometrical relation associated with (11) can be encapsulated in the state estimation procedure in order to improve the estimation quality.

## 4. Localization-Oriented Control Law

In this section, a motion control scheme for range-based relative localization is proposed. Using this strategy, it is possible to infer the relative velocity of agents from the motion measurements. Consider the following control law for each vehicle:

(15) $$\begin{array}{l} {\text{v}}_{i}\left(t\right)=\underset{j\in N_{i}}{\sum }K\left({\text{x}}_{i}\left(t\right)-x_{j}\left(t\right)\right)\;:\;K>0,\;i\;\in \;V \end{array}$$

where *K* ∈ ℝ is a positive constant. According to such schema, the motion of each vehicle *i* depends only on the relative positions with its neighbors, namely **z**_*ij*_ with $j\in N_{i}$.

It is worth noting that in our framework Equation (15) cannot be directly implemented (as the actual relative positions **z**_*ij*_ are not known to vehicle *i*), but we replace the estimated relative positions ${\hat{\text{z}}}_{ij}$ instead:

(16) vi(t)=K∑j∈Niz^ij(t), i∈V,

leading to the following relative velocities:

(17) vij(t)=K(∑h∈Niz^ih(t)-∑ρ∈Njz^jρ(t))  :  (i,j)∈E

Details about the specific computation of the estimated relative positions z^ij to be used in (16) are provided in the next section.

When agents adopt this control law, the system (5–6) becomes:

(18) z∙ij(t)=K(∑h∈Niz^ih(t)-∑ρ∈Njz^jρ(t)),(i,j)∈E

(19) $$\begin{array}{l} y_{ij}\left(t\right)=\|{\text{z}}_{ij}\left(t\right){\|}^{2}. \end{array}$$

As a final remark, it is interesting to note that the control law in Equation (15) has the same structure of a consensus protocol as those described in Olfati-Saber and Murray (2004) and Ren and Beard (2007). Control strategies based on such kind of protocols have been widely studied for the coordination or formation control of a team of agents (Leonard et al., 2007; Ren and Cao, 2010). In this paper, we do not seek control objectives but we rather use Equation (16) as a localization-oriented control protocol that each vehicle must follow at each *t* = *kT*_*s*_, being *T*_*s*_ a fixed time interval. Therefore, within each interval the agents keep their velocity constant. Indeed, in this paper such control law is adopted to make the motion informative of each agent's position and velocity, and hence make the range-based relative localization of a networked group of underwater vehicles solvable in finite time so that it can be executed as a routine inside a mission when localization is needed. This is useful when, during a cooperative mission, the relative localization accuracy of the agents decreases; in this case, the proposed localization-oriented control law can be activated so as to improve the accuracy of the relative localization.

The advantage of such solution with respect to the work presented in De Palma et al. (2015) is that by adopting the motion control scheme for range-based relative localization in (16), there is no need for the agents to share their velocity information in the communication channel. Indeed, an agent is able to derive the velocity of the other agents from the knowledge of the adopted control law and the estimated relative positions. This result in a significant reduction of the communication load over the network. The results achieved in this paper are particularly relevant in underwater applications where the bandwidth is often limited due to the acoustic communications.

## 5. Observer Design

The estimation of the relative positions **z**_*ij*_(*t*) in Equations (18), (19) is tackled resorting to the methods presented in Indiveri et al. (2016). Let us integrate Equation (18)

(20) zij(t) = zij(t0)+∫t0tK(∑h∈Niz^ih(τ)-∑ρ∈Njz^jρ(τ))dτ            = zij(t0)+dij(t),

with **d**_*ij*_(*t*) defined as

(21) dij(t):=∫t0tK(∑h∈Niz^ih(τ)-∑ρ∈Njz^jρ(τ))dτ∈ℝ3×1.

Equation (20) allows to compute

(22) $$\begin{array}{l} {\text{z}}_{ij}^{⊤}\left(t_{0}\right){\text{z}}_{ij}\left(t_{0}\right)=y_{ij}\left(t_{0}\right)=\\ \;=y_{ij}\left(t\right)+\|{\text{d}}_{ij}\left(t\right){\|}^{2}-2{\text{d}}_{ij}^{⊤}\left(t\right){\text{z}}_{ij}\left(t\right) \end{array}$$

yielding

(23) $$\begin{array}{l} {ȳ}_{ij}\left(t\right):=\frac{1}{2}\left[y_{ij}\left(t\right)-y_{ij}\left(t_{0}\right)+\|{\text{d}}_{ij}\left(t\right){\|}^{2}\right] \end{array}$$

(24) $$\begin{array}{l} {ȳ}_{ij}\left(t\right)={\text{d}}_{ij}^{⊤}\left(t\right){\text{z}}_{ij}\left(t\right). \end{array}$$

The term ȳ_*ij*_(*t*) defined in Equation (23) as well as the term **d**_*ij*_(*t*) defined in (21) are both known quantities, so that the new linear output equation in Equation (24) can be considered. Consequently, the original non-linear model (Equations 5 and 6) can be expressed as a Linear Time-Varying (LTV) model

(25) z∙ij(t)=K(∑h∈Niz^ih(t)-∑ρ∈Njz^jρ(t))

(26) $$\begin{array}{l} {ȳ}_{ij}\left(t\right)={\text{d}}_{ij}^{⊤}\left(t\right){\text{z}}_{ij}\left(t\right). \end{array}$$

Thus, the estimation of **z**_*ij*_(*t*) in (25, 26) can be addressed exploiting the standard linear system theory. It should be noticed that the output matrix ${\text{d}}_{ij}^{⊤}\left(t\right)$ of the LTV model depends on the control input, hence the observability depends on the agents' relative velocity **v**_*ij*_. It can be proven that a sufficient condition for the observability of the original system (5–6) on [*t*_0_, *t*] is the invertibility of the observability Gramian of the LTV system (25, 26) defined as:

(27) $$\begin{array}{l} G\left(t_{0},t\right)=\int_{t_{0}}^{t}{\text{d}}_{ij}\left(\tau \right){\text{d}}_{ij}^{⊤}\left(\tau \right)d\tau . \end{array}$$

The reader should refer to Indiveri et al. (2016) for a detailed analysis of the observability properties of such a system. Let us consider the discrete time formulation of the LTV system given by:

(28) zij(k+1)=zij(k)+K(∑h∈Niz^ih(k)-∑ρ∈Njz^jρ(k))Ts+ω(k)

(29) $$\begin{array}{l} {ȳ}_{ij}\left(k\right)={\text{d}}_{ij}^{⊤}\left(k-1\right){\text{z}}_{ij}\left(k\right)+\epsilon \left(k\right) \end{array}$$

with

(30) $$\begin{array}{l} {ȳ}_{ij}\left(k\right)=\frac{1}{2}\left[y_{ij}\left(k\right)-y_{ij}\left(0\right)+\|{\text{d}}_{ij}\left(k-1\right){\|}^{2}\right], \end{array}$$

(31) dij(k-1)=∑l=0k-1K(∑h∈Niz^ih(l)-∑ρ∈Njz^jρ(l))Ts,

where ***ω***(*k*) and ϵ(*k*) are assumed to be zero mean, Gaussian, white, and uncorrelated process and measurements noises with covariances ***Q***(*k*) and *R*(*k*), respectively, and *T*_*s*_ represents the sampling time. A standard Kalman filter can be applied to the model in Equations (28) and (29), leading to the following equations:

(32) $${\hat{\text{z}}}_{ij}(k+1|k)={\hat{\text{z}}}_{ij}(k|k)+K\left(\sum_{h\in N_{i}}{\hat{z}}_{ih}(k)-\sum_{\rho \in N_{j}}{\hat{\text{z}}}_{j\rho }(k)\right)T_{s}$$

(33) $$P_{ij}(k+1|k)=P_{ij}(k|k)+\;Q(k)$$

(34) $$\begin{array}{l} K=(P_{ij}^{-1}(k+1|k)\\ \;+\;{\text{d}}_{ij}(k)R{\left(k+1\right)}^{-1}{\text{d}}_{ij}^{⊤}(k){)}^{-1}{\text{d}}_{ij}(k)R{\left(k+1\right)}^{-1} \end{array}$$

(35) $$\begin{array}{l} {\hat{z}}_{ij}(k+1|k+1)={\hat{z}}_{ij}(k+1|k)+K(\bar{y}(k+1)\\ \;-{\text{d}}_{ij}^{⊤}(k)\;z_{ij}(k+1|k)) \end{array}$$

(36) $$\begin{array}{l} P_{ij}(k+1|k+1)=(P_{ij}^{-1}(k+1|k)\\ \;+\;{\text{d}}_{ij}(k)R{\left(k+1\right)}^{-1}{\text{d}}_{ij}^{⊤}(k){)}^{-1}. \end{array}$$

In the considered scenario, thanks to the intra-vehicles acoustic communications, each agent is able to know the estimations z^ij and their covariances ***P***_*ij*_. Therefore, each agent can improve the estimation accuracy exploiting the additional geometric constraints (11). To this aim, we can benefit from the projection approach described in the following subsection.

### 5.1. Constraint Exploitation for the Estimate Improvement

Assuming to know the *m* Kalman filter estimates z^ij, it is possible to incorporate the constraint (11) in the estimation framework resorting to the approach described in Simon (2006). Let us define the Kalman filter estimate z^* as

(37) z^*(k)=(⋯z^ij⊤(k)⋯)⊤∈ℝ3m : (i,j)∈E,

and its posterior covariance as

(38) $$\begin{array}{l} P^{*}\left(k\right)=\text{diag}\left(\cdots P_{ij}\left(k\right)\cdots \;\right)\in {\mathbb{R}}^{3m\times 3m}\;:\left(i,j\right)\in E. \end{array}$$

An estimate z^p* satisfying the constraint (11) can be derived projecting the Kalman filter estimate onto the constraint surface; this would lead to the following solution:

(39) z^p*(k)=U z^*(k)

where ***U*** is the projection operator

(40) $$\begin{array}{l} U:=I_{3m\times 3m}-\left[W^{-1}D^{⊤}{\left(DW^{-1}D^{⊤}\right)}^{-1}\right]D \end{array}$$

such that $U^{2}=U,DU=0_{3\left(m-n+1\right)\times 3m}$, and ***W*** ∈ ℝ^3*m*×3*m*^ is any positive definite weighting matrix. As proven in Simon and Chia (2002), if the weight matrix ***W*** in Equation (40) is chosen as ***W*** = P^*−1^, then the estimate z^p* in Equation (39) is minimum variance, namely

(41) $$\begin{array}{l} P_{p}^{*}\leq P^{*} \end{array}$$

being $P_{p}^{*}$ the error covariance of z^p*; however, if the weight matrix ***W*** is chosen as ***W*** = ***I***, then the constrained estimate z^p* in Equation (39) is always closer to the true state than the unconstrained estimate, namely

(42) ‖z^*-z^p*‖≤‖z^*-z^*‖.

Choosing the weight matrix ***W*** = P^*−1^(*k*), the estimate z^p*(k) becomes:

(43) z^p*(k)=z^*(k)-[P*(k)D⊤(DP*(k)D⊤)-1]Dz^*(k),

and resulting posterior covariance is given by

(44) $$\begin{array}{l} P_{p}^{*}\left(k\right)=P^{*}\left(k\right)-P^{*}\left(k\right)D^{⊤}{\left(DP^{*}\left(k\right)D^{⊤}\right)}^{-1}DP^{*}\left(k\right). \end{array}$$

From the z^p* resulting from (39), it is possible to extract the single z^ij that appears in the control and estimation equations (16, 17, 18, 20, 21, 28, 25, 31, 32). Hence, the minimum variance estimate z^p* is actually used by each agent to set its velocity according to the control law (Equation 16). Therefore, we assume that this velocity is kept constant for the fixed time interval *T*_*s*_, namely it does not change until a new estimation is available.

### 5.2. Remark

It is worth noting that the output ȳ_*ij*_(*t*) defined in Equation (23) depends on the first measurement *y*_*ij*_(*t*_0_). This dependency may affect the robustness of the solution as a single erroneous measurement (e.g., an outlier or a fault signal) at *t* = *t*_0_ would jeopardize the output. This issue can be overcome by periodically resetting the measurement *y*(*t*_0_) with *y*(*t*). This would also prevent possible uncertainties in the knowledge of ***v***_*ij*_(*t*) from causing an unbounded bias in the displacement **d**_*ij*_(*t*) in Equation (23) used to compute ȳ_*ij*_(*t*). In the discrete time case, this would correspond to periodically mapping $y_{ij}\left(0\right)\to y_{ij}\left(k^{*}\right)$ as if the measurement had started at step k^*^ while the state estimate z^ij(k+1|k+1) follows its update dynamics. The results presented in the following section refer to the discrete time case with periodic mapping of the initial measurement *y*_*ij*_(0) with *y*_*ij*_(*k*−1) (i.e., k^*^ = *k*−1). Consequently, the displacement in Equation (31) becomes ${\text{d}}_{ij}\left(k-1\right)=\overset{k-1}{\underset{l=k^{*}}{\sum }}{\text{v}}_{ij}\left(l\right)T_{s}={\text{v}}_{ij}\left(k-1\right)T_{s}$.

## 6. Delays in Range Measurements Acquisition

One key point to have in mind when dealing with underwater networks is that acoustic communications may be subject to relatively large delays. In particular, communication latency is due to the physics of the communication channel as well as to the specific networking protocol employed. This latter component of the delay may be eventually reduced accepting higher packet loss probabilities. While details about the assessment of the communication latency are not addressed in this work, it should be noted that delays may be significant for larger distances and should be accounted for in the estimation framework. Indeed, this is the case within the approach described in this work where the delay needs to be known.

In this framework, the range measurements available during each step of the estimation process will be *y*_*ij*_(*t*−τ_*ij*_), rather than *y*_*ij*_(*t*), having denoted with τ_*ij*_ the time delay in the measurement acquisition due to the acoustic communication network. This arises the problem of how it is possible to obtain the actual range *y*_*ij*_(*t*) from the knowledge of the delayed measurement *y*_*ij*_(*t*−τ_*ij*_), and the time delay τ_*ij*_, in order to properly perform the observer for the relative position estimation **z**_*ij*_. Let consider the intra-vehicle range *y*_*ij*_(*t*):

(45) $$\begin{array}{l} y_{ij}\left(t\right)={\text{z}}_{ij}{\left(t\right)}^{⊤}{\text{z}}_{ij}\left(t\right) \end{array}$$

and its time derivative:

(46) $$\begin{array}{l} {ẏ}_{ij}\left(t\right)=2\;\overset{∙}{\text{z}}{\left(t\right)}_{ij}^{⊤}\;{\text{z}}_{ij}\left(t\right)=2\;{\text{v}}_{ij}{\left(t\right)}^{⊤}\;{\text{z}}_{ij}\left(t\right) \end{array}$$

Equation (46) allows computing *y*_*ij*_(*t*) from the knowledge of *y*_*ij*_(*t*−τ_*ij*_) and τ_*ij*_ as:

(47) $$\begin{array}{l} y_{ij}\left(t\right)=y_{ij}\left(t-\tau_{ij}\right)+\int_{t-\tau_{ij}}^{t}2\;{\text{v}}_{ij}{\left(\tau \right)}^{⊤}{\text{z}}_{ij}\left(\tau \right)d\tau \end{array}$$

Exploiting Equation (47), time delays in the measurements are taken into account mitigating their effects on the estimation process. It is worth highlighting that the sampling time *T*_*s*_ of the Kalman filter should be properly chosen.

As a final remark, it is worth noting that it is not possible to implement Equation (47) as it is because the actual relative positions **z**_*ij*_ are not known, and we use the current estimations z^ij instead:

(48) yij(t)=yij(t-τij)+∫t-τijt2 vij(τ)⊤z^ij(τ)dτ.

The numerical integration of Equation (48) leads to the following discrete-time equation:

(49) yij(k)≈yij(kTs-τij)+∑l=0(τij/dT)2 vij(kTs-τij+ldT)⊤ z^ij(kTs-τij+ldT)dT.

where *dT* denotes the integration time. Notice that in spite of the lack of an analytical proof of convergence of Equation (49) to the true measurement *y*_*ij*_, all the numerical results confirm the effectiveness of this approach.

The overall control and estimation procedure is illustrated in Algorithm 1. Summarizing, at each time step the last available constrained estimates of **z**_*ij*_ are used by the control law of each vehicle using Equation (16). Then, the measurements *y*_*ij*_ are acquired: in case of delays, the current *y*_*ij*_ is estimated through Equation (49). Finally, the observer updates the estimates of the variables **z**_*ij*_ using the constrained Kalman filter solution.

**Algorithm 1:** Control and estimation algorithm

**Require:** z^ij(k|k),Pij(k|k),z^pij(k|k),Q(k),R(k),yij(0), $y_{ij}\left(\left(k+1\right)T_{s}-\tau_{ij}\right),\tau_{ij}:\left(i,j\right)\in E$
**Ensure:** z^ij(k+1|k+1),Pij(k+1|k+1),z^p(k+1|k+1),Ppij(k+1|k+1)
1: vi(k)←K∑h∈Niz^pih(k|k)
2: vij(k)←K(∑h∈Niz^pih(k|k)-∑ρ∈Njz^pjρ(k|k))
3: ${\text{d}}_{ij}\left(k\right)\leftarrow \overset{k}{\underset{l=0}{\sum }}{\text{v}}_{ij}\left(l\right)T_{s}$
4: **if** τ_*ij*_≠0
5: compute *y*_*ij*_(*k*+1) from (50)
6: **end**
7: ${ȳ}_{ij}\left(k+1\right)\leftarrow \frac{1}{2}\left[y_{ij}\left(k+1\right)-y_{ij}\left(0\right)+\|{\text{d}}_{ij}\left(k\right){\|}^{2}\right]$
8: compute the KF estimation using (33-37): z^ij(k+1|k+1),Pij(k+1|k+1):(i,j)∈E
9: identify independent geometric constraints in terms of ***D***
10: project KF estimation on constraint equations using (43-44)
11: **return** z^ij(k+1|k+1),Pij(k+1|k+1),z^p(k+1|k+1),Ppij(k+1|k+1):(i,j)∈E

## 7. Communication Protocols

In this section, we describe the communication policy that we adopted to perform the range data exchange among the agents during the intersampling period. This step is instrumental to make the computation of Equations (32), (36) possible at each sampling time, and in turn the projection (Equations 43, 44).

Several approaches are possible; we propose two alternative solutions, which we refer to as *centralized* approach and *decentralized* approach. In the centralized approach, only one agent, a *leader* agent, is expected to perform the computation of Equations (32)–(33) and (43), (44) so that the communication policy is organized in order to make the data flow to the leader for the twofold task of reconstructing the topology of the RPMG established and collecting a complete set of range measurement to perform the estimation of Equations (32)–(36). If necessary or useful, the leader agent sends back the resulting estimated positions among the agents using the same scheme reversed. In the decentralized approach, all agents have the capability of performing the computation of (32)–(36) and (43), (44) and hence the communication policy is oriented to spread the range data among agents to distribute them to all, so that each agent performs the computation of the positions estimation. It is worth noting that the term centralized/decentralized is related to the computation of Equations (32)–(36), and hence to the fact that the “holder” of the estimated value is only one agent or any one of the network.

Regardless of the strategy adopted, an issue to consider is that the RPMG cannot be known in advance, and the topology identification of it is instrumental to the computation of Equations (32)–(36). In this respect, we assume that the number of the vehicles *n* and a preassigned labeling of the agents is known in advance, while the connection topology is unknown to any vehicle and it must be reconstructed as well using any approach.

We assume that all agents involved are equipped with synchronized clocks so as to use One-Way Travel Time (OWTT) range measurement schemas. Then, a Time-Division Multiple Access (TDMA) scheme can be employed to access the communication channel. Under this hypothesis, the communication among agents is unidirectional; this choice is conservative in order to avoid the chance of packet collisions and the management of the resulting loss of data. It should be noted that the duration of the time slots depends on the available bit rate and on the specific communication protocol. Examples of acoustic sensors commonly used in underwater environment are the middle frequency (MF) modems (18–34 kHz) by Evologics (Kebkal et al., 2017). They have been recently used for underwater positioning purposes during geotechnical survey experiments performed within a European project (Abreu et al., 2016). Such modems are characterized by a nominal bit rate in the range 3.10–3.85 kbps, hence compatible with the application at hand.

It is now worth mentioning that the amount of time needed for two agents to communicate using acoustic signals may be significant for large distances and in this paper it is accounted for as described in section 6. Indeed, sound speed underwater is approximately 1500 m/s, namely about six orders of magnitude lower than the speed of electromagnetic signals in air.

**Figure 2:**
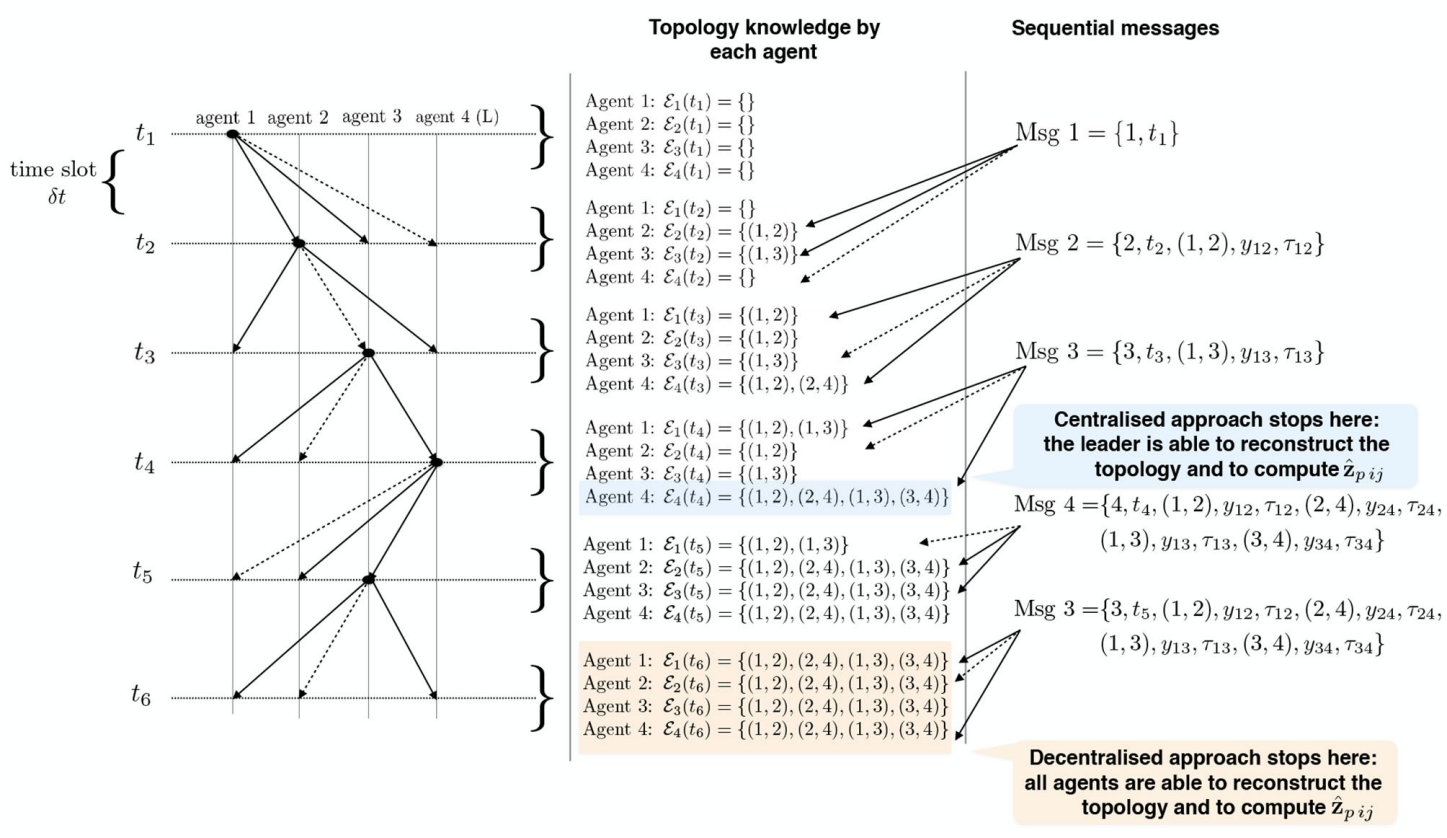
Example of acoustic interrogation schema for acquiring range measurements and identify the connection topology for Relative Position Measurement Graph (RPMG) in Figure 3.

**Figure 3:**
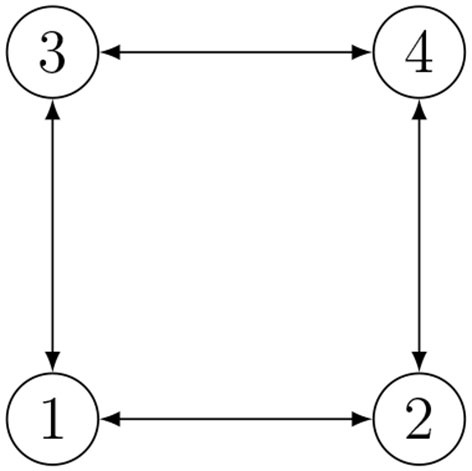
Example of Relative Position Measurement Graph (RPMG) with 4 nodes and 4 edges.

**Figure 4:**
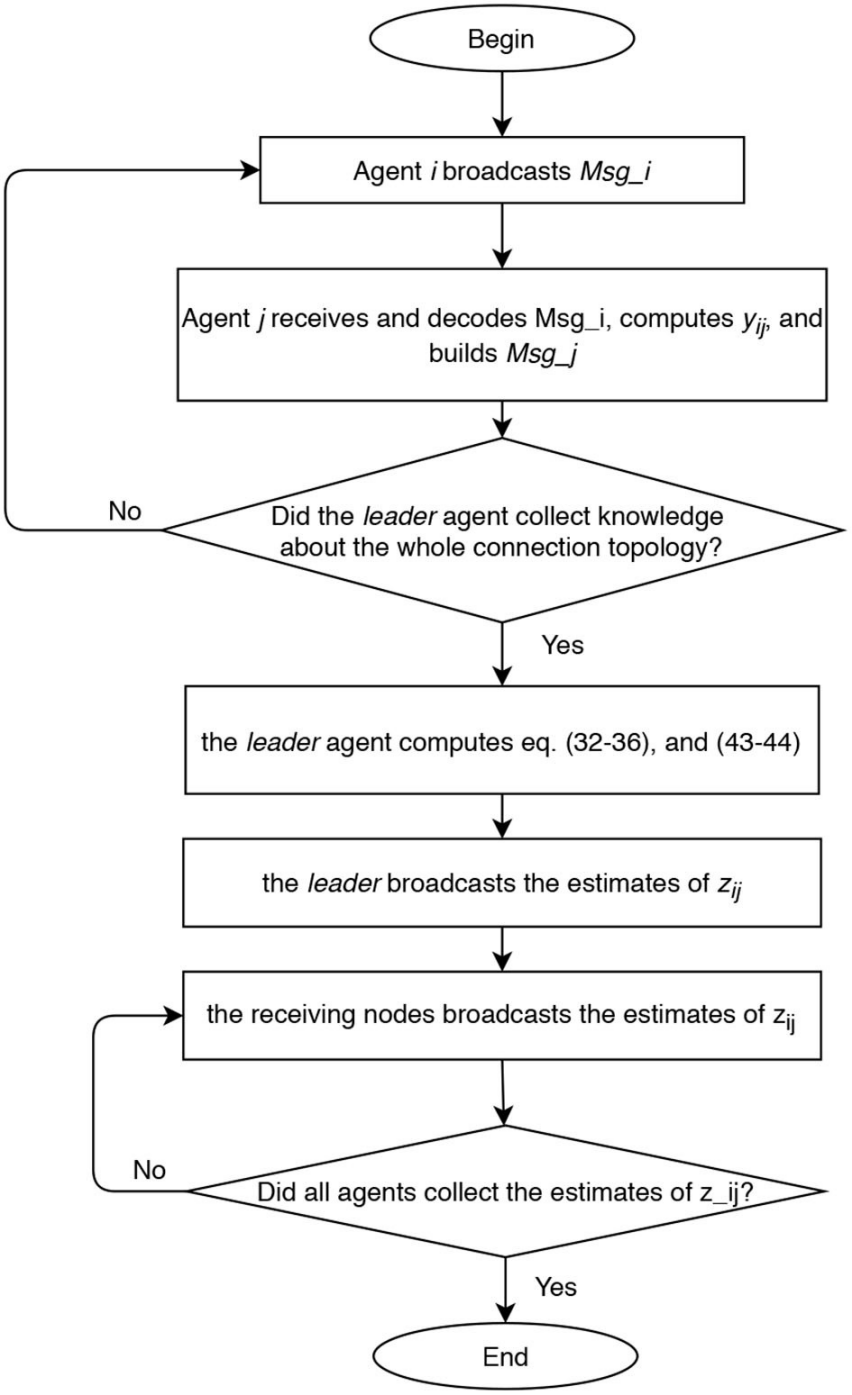
Flow chart of centralized approach for range measurements acquisition and relative positions estimation.

**Figure 5:**
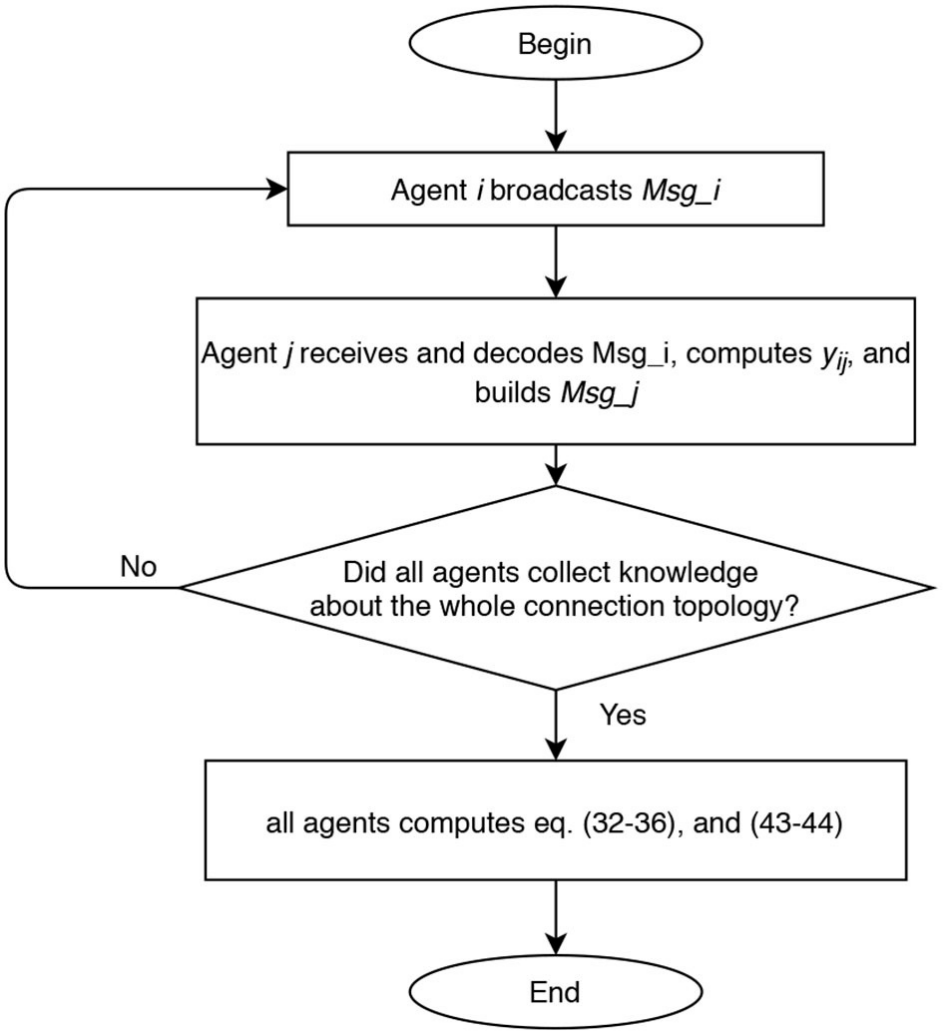
Flow chart of decentralized approach for range measurements acquisition and relative positions estimation.

According to all the previous considerations, we established our communication policy under the following assumptions. We refer to Figure 2 as to a description of the idea in the case of *n* = 4 and RPMG as in Figure 3. The two strategies are put in a pseudo-algorithm form (in the form of a flowchart) depicted in Figures 4, 5.

We assume that vehicles are organized to send packets one by one. The agents are labeled from the beginning and they follow their labeling in order to send broadcast packets according to the agreed protocol (which depends on the type of approach, this is detailed in the following). Each packet is received only by the neighboring agents and it takes a non-zero travel time to reach the receiver, so we set equal to δ*t* the maximum travel time (which depends on the sensor range and environmental parameters). All agent are aware of the starting time of the estimation procedure, say $\bar{t}$. Agent *i* sends its packet at time $\bar{t}+\left(i-1\right)\cdot \delta t$ and this packet reaches the agent *j*, $j\in N_{i}$ within the time span $\left(\bar{t}+\left(i-1\right)\cdot \delta t,\bar{t}+i\cdot \delta t\right)$. This is periodically repeated at each $\bar{t}+\kappa T_{s}$.

In the centralized approach, the range data that are needed to run the filter can be distributed in the team of *n* members as illustrated in Figure 4: agents sequentially (one in each time slot) broadcast a data packet containing their identifying label, a time stamp, all the edges it is aware of, and all the corresponding range data and measurements delays. After all agents but one (i.e., after *n*−1 time slots) execute the protocol, the leader agent collects knowledge about the whole connection topology. Hence, the leader agent knows all the information, i.e., relative distances (6), required to solve the estimation problem taking into account the additional geometric constraints (11) associated with the connection topology. Once computed the estimates z^p* of the *m* relative positions using the collected information, the leader agent broadcasts to all agents a data packet containing such estimates. Overall, 2*n* − 2 time slots are required to complete one estimation step. This kind of scaling appears to be most likely acceptable for most applications involving a limited number of vehicles.

In the decentralized approach, considering that the communication graph is unknown to the agents, the communication policy is implemented with the aim of retrieving the graph topology and spreading the range data to all agents. Each agent during its time slot broadcasts a data packet containing its label, a time stamp, the set of links already identified, and the corresponding range data and measurements delays. All agents receiving the ping, decode the data packet, and identify the link between itself and the transmitter agent. This is repeated until all agents collect knowledge about the whole connection topology. In the worst case, 2*n* − 2 communications slots are required to ensure that all agents have identified the connection topology. At this point, each agent can perform the estimation z^p* of the relative positions.

The main differences between the two approaches can be deduced by the schemes in Figures 4, 5, and we briefly comment them in the following. In the centralized approach, only one agent perform the elaboration, and it can be useful when the team is heterogeneous and some agents have higher computational capacity than others. However, the centralized approach requires a larger amount of communicated data when the estimated state is transmitted to all agents.

## 8. Simulations

The proposed range-based mutual localization for a team of underwater vehicles is here tested on the RPMG in Figure 3 relative to a group of *n* = 4 agents and *m* = 4 communication links. The corresponding geometric constraints are as follows:

(50) $$\begin{array}{l} D{\text{z}}^{*}=\left[\begin{array}{cccc} I_{3\times 3} & I_{3\times 3} & I_{3\times 3} & -I_{3\times 3} \end{array}\right]\left[\begin{array}{c} {\text{z}}_{12}\\ {\text{z}}_{24}\\ {\text{z}}_{43}\\ {\text{z}}_{13} \end{array}\right]=0_{3\times 1}. \end{array}$$

The velocity inputs of each agent are assigned according to the localization-oriented control law in Equation (16) with *K* = 0.1:

$$\begin{array}{l} v_{1}(k)=-K(z_{12}(k)+z_{31}(k));\\ v_{2}(k)=-K(z_{12}(k)+z_{24}(k));\\ v_{3}(k)=-K(z_{31}(k)+z_{43}(k));\\ v_{4}(k)=-K(z_{24}(k)+z_{43}(k)). \end{array}$$

**Figure 6:**
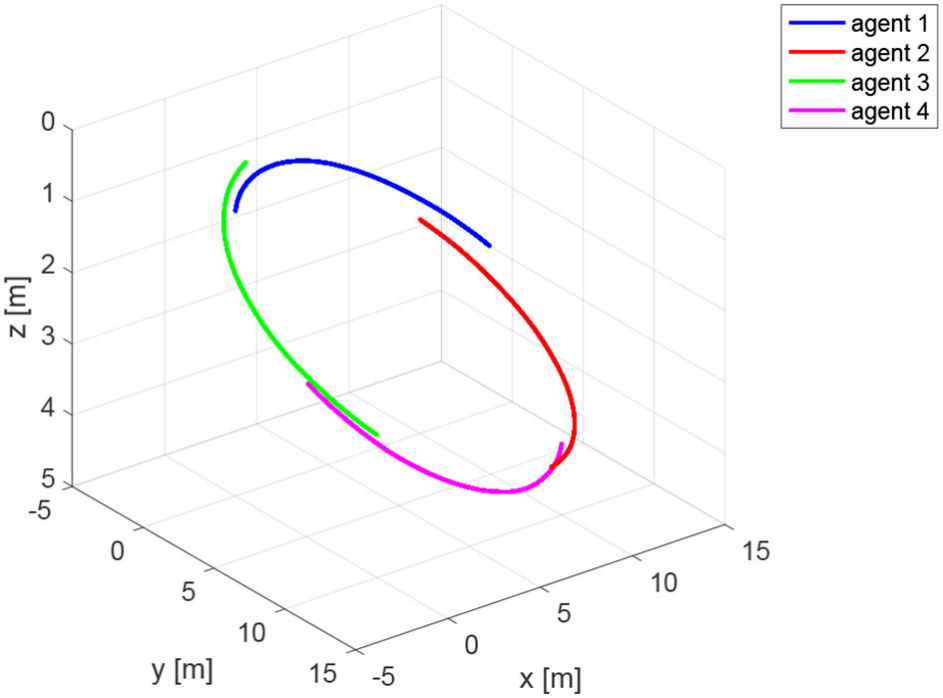
Trajectories of the agents for the simulation based on Relative Position Measurement Graph (RPMG) in Figure 3.

**Figure 7:**
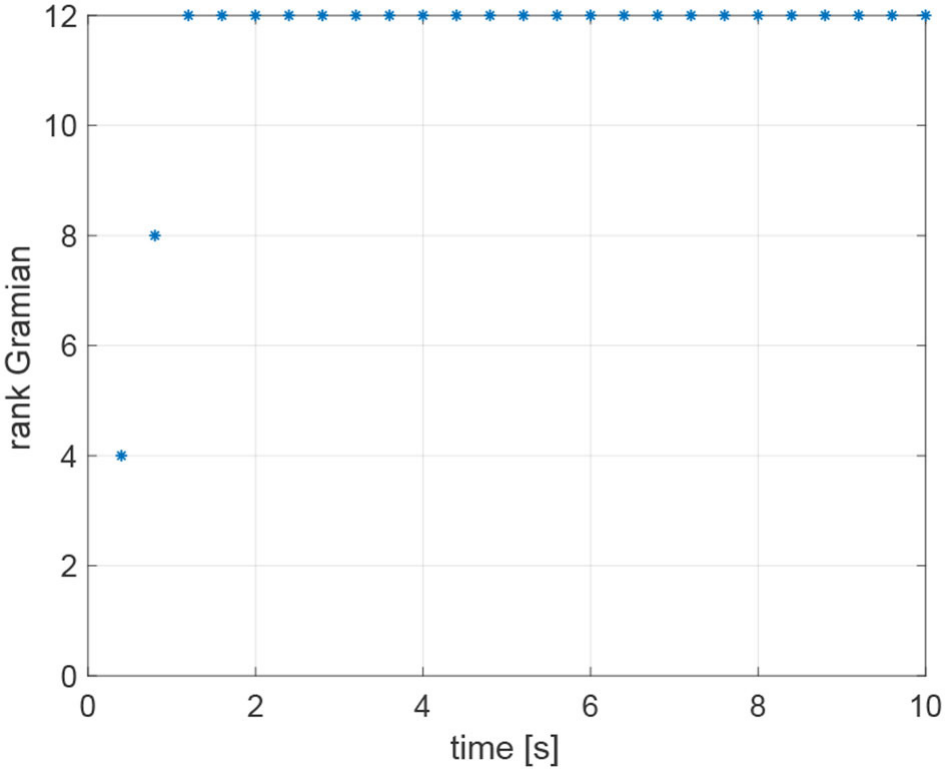
Rank of the observability Gramian for the simulation based on Relative Position Measurement Graph (RPMG) in Figure 3.

The agents are located in the following initial positions: ${\text{x}}_{1}\left(0\right)={\left(0,0,1\right)}^{⊤}\text{m}$; ${\text{x}}_{2}\left(0\right)={\left(10,0,2\right)}^{⊤}\text{m}$; ${\text{x}}_{3}\left(0\right)={\left(0,10,3\right)}^{⊤}\text{m}$; ${\text{x}}_{4}\left(0\right)={\left(10,10,4\right)}^{⊤}\text{m}$. Without loss of generality, the range measurements are assumed to be acquired with different time delays τ_*ij*_, namely τ_12_ = 0.1s, τ_24_ = 0.2s, τ_31_ = 0.3s, *andτ*_43_ = 0.4s, whereas a sampling time *T*_*s*_ = 0.4*s* has been considered. At each sampling time *T*_*s*_, the actual range *y*_*ij*_(*t*) is derived from the knowledge of the delayed measurement *y*_*ij*_(*t*−τ_*ij*_), and the time delay τ_*ij*_ according to Equation (49). The resulting trajectories are shown in Figure 6. It is worth remarking that the proposed agents velocities *v*_*i*_ guarantee the observability of the system (Equations 25 and 26). Indeed, it can be verified, by direct calculation, that the motion generated by the control law (16) verifies the full rank condition on the observability Gramian (27) of the system, i.e., rank(*G*) = 3*m* = 12. Figure 7 shows the rank of the Gramian along the trajectory, and a few range acquisitions are needed to get a full rank Gramian matrix. Therefore, given the observability of the system, the states **z**_*ij*_ can be estimated using the Kalman observer in Equations (32)– (36), with covariance ***Q*** = 0.910^−5^·diag(1, 1, 1)m^2^, covariance *R* = 0.25m^2^, and initial condition given by

(51) z^ij(0)~N(zij(0),Pij(0)), Pij(0)=4·diag(1,1,1)m2.

**Figure 8:**
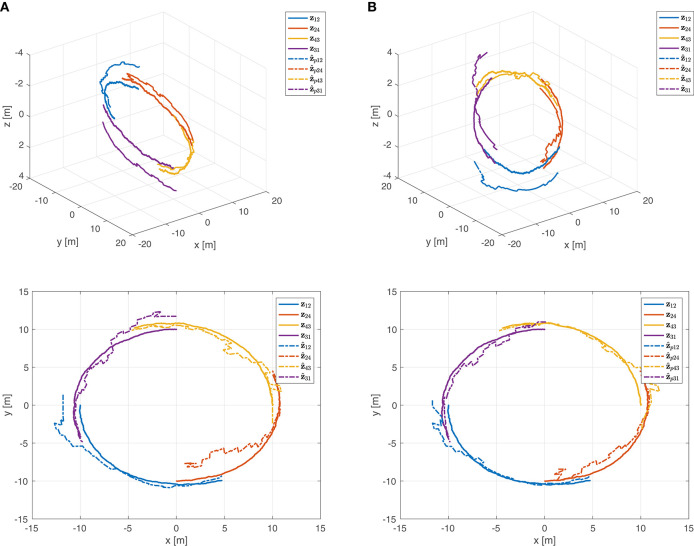
**(A)** Estimation of the relative motions in 3D (top) and 2D (bottom). **(B)** Constrained estimation of the relative motions in 3D (top) and 2D (bottom).

**Figure 9:**
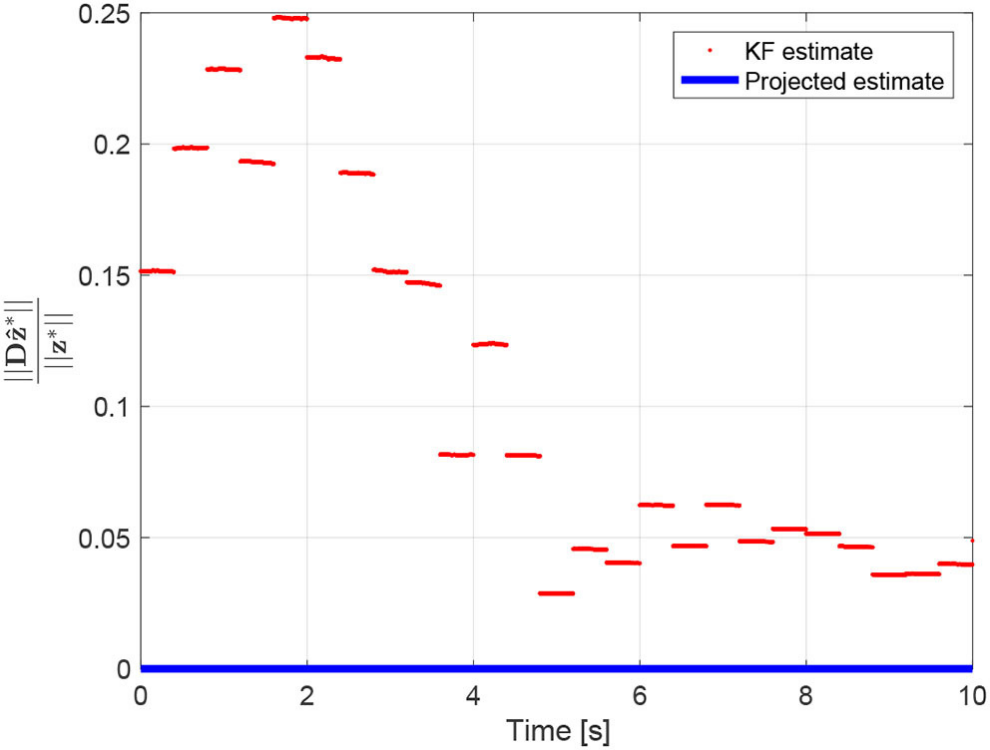
Equality constraint ‖Dz^*‖‖z*‖=0.

**Figure 10:**
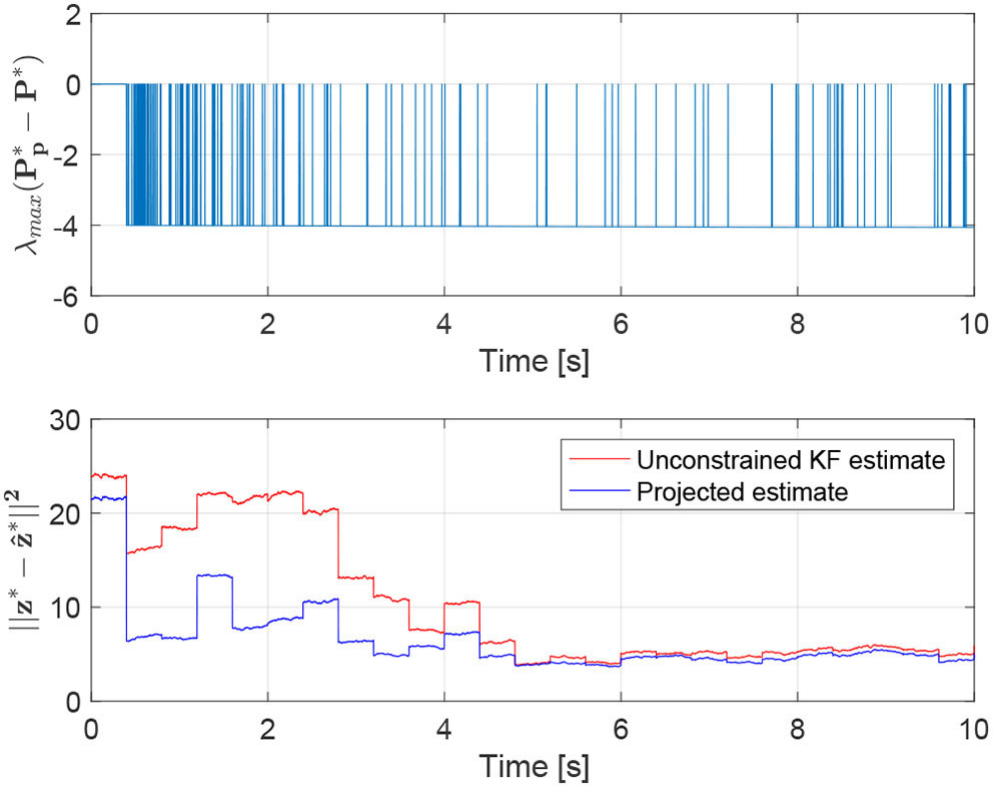
Maximum eigenvalue of the matrix $P_{p}^{*}\left(k\right)-P^{*}\left(k\right)$ **(Top)** and norm of the estimation errors **(Bottom)**.

The estimate z^*=(z^12 ⊤z^24 ⊤z^43 ⊤z^13⊤)⊤∈ℝ12 obtained using the Kalman filter is reported in Figure 8A. This estimate violates the equality constraint (50). A constrained state estimate can be obtained projecting the standard Kalman filter estimate z^* onto the constraint surface through Equations (43), (44). This leads to the projected estimation illustrated in Figure 8B. Figure 9 reports the norm of the equality constraints, ‖Dz^*‖‖z*‖. It is worth noting that the unconstrained Kalman estimate (red line) does not satisfy exactly the constraint, whereas the constrained Kalman estimate (blue line) satisfies the equality constraint, namely ‖Dz^*‖‖z*‖=0. Moreover, as expected, the constrained estimate is also characterized by a reduced covariance, i.e., $P_{p}^{*}-P^{*}<0$. Indeed, the maximum eigenvalue of the matrix $P_{p}^{*}-P^{*}$, shown in Figure 10, is always negative (not positive), confirming the improvement obtained by exploiting the additional information provided by the geometric constraints.

Note that regarding the norm of the estimation error, even if the weight ***W*** is chosen as ***W*** = P^*−1^(*k*) rather than ***W*** = ***I***, the projected estimates still provide better results with respect to the corresponding unconstrained Kalman estimates. The norm of the estimation error for both estimates is reported in Figure 10.

**Figure 11:**
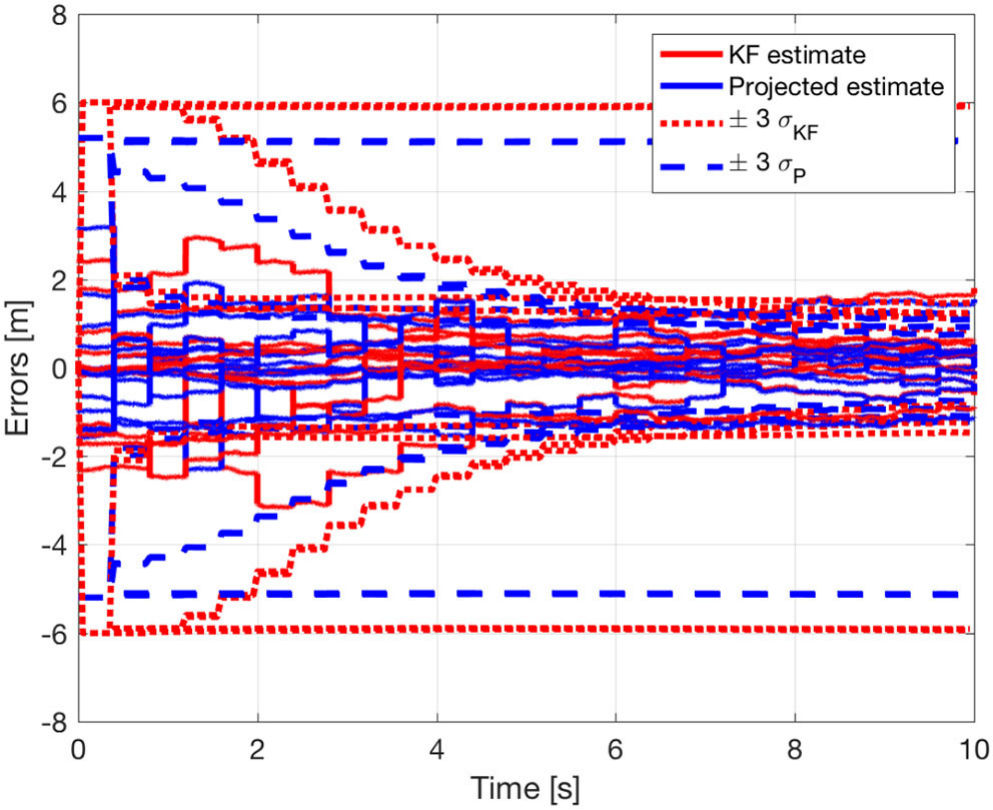
Estimation errors and uncertainty regions for the relative positions estimations z^*^.

**Figure 12:**
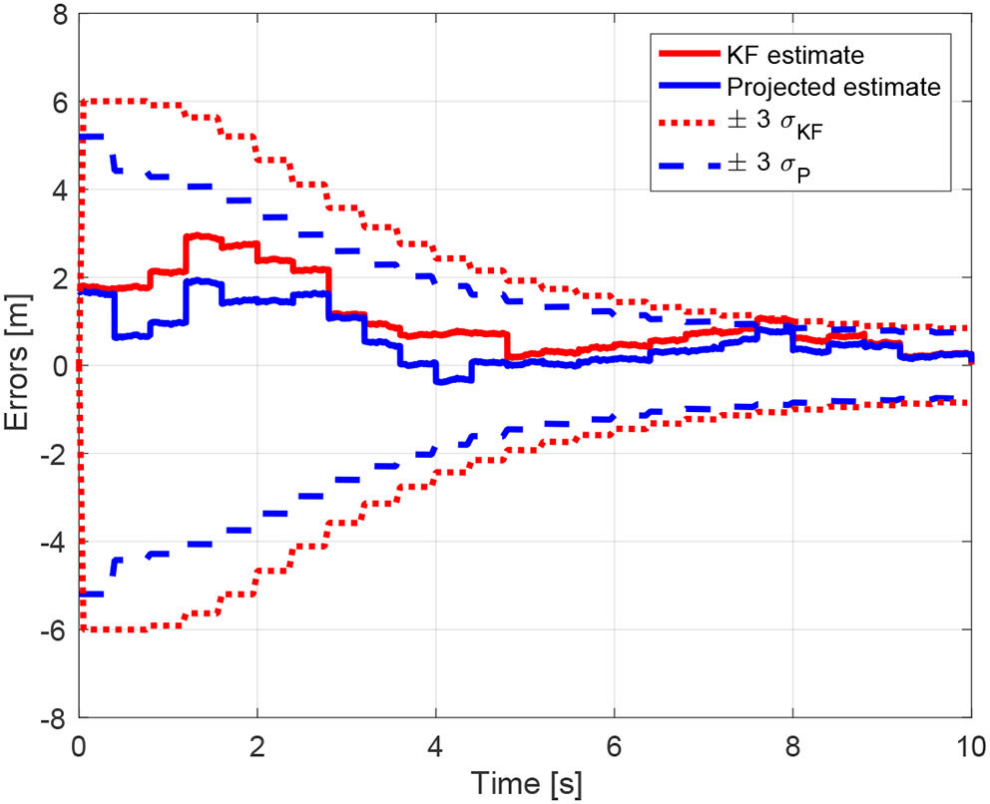
Estimation errors and uncertainty regions for the relative positions estimations of the first component of z^*^.

The benefits of including the geometric constraints into the estimation framework are more evident in Figure 11, where the estimation error and the corresponding uncertainty region of each component of the state z^*^ ∈ ℝ^12^ are shown for both estimates, unconstrained and constrained. The estimation errors and the uncertainty region for the first component of the state z^*^ ∈ ℝ^12^ are also depicted in Figure 12. It is interesting to note that, as already highlighted, the uncertainty region of the constrained estimates is smaller than the one related to the standard Kalman estimate. As a final remark, it is worth noting that the estimates in Figures 8, 11, 12, as well as the equality constraint in Figure 9, have sharp leaps whenever a range update is processed, i.e., every *T*_*s*_ seconds. Moreover, the management of the delays in range measurements as described in section 6 allows to correctly process the measurements without compromise the convergence of the estimations.

**Figure 13:**
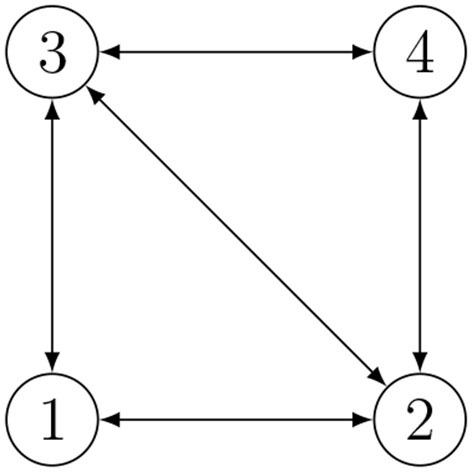
RPMG with *n* = 4 agents and *m* = 5 communication links.

A further simulation is undertaken on the more complex RPMG illustrated in Figure 13 relative to a group of *n* = 4 agents and *m* = 5 communication links. The velocity inputs of each agent are assigned according to the localization-oriented control law in Equation (17) with *K* = 0.1:

$$\begin{array}{l} v_{1}(k)=-K(z_{12}(k)+z_{31}(k));\\ v_{2}(k)=-K(z_{12}(k)+z_{24}(k)+z_{32}(k));\\ v_{3}(k)=-K(z_{31}(k)+z_{43}(k)+z_{32}(k));\\ v_{4}(k)=-K(z_{24}(k)+\;z_{43}(k)). \end{array}$$

**Figure 14:**
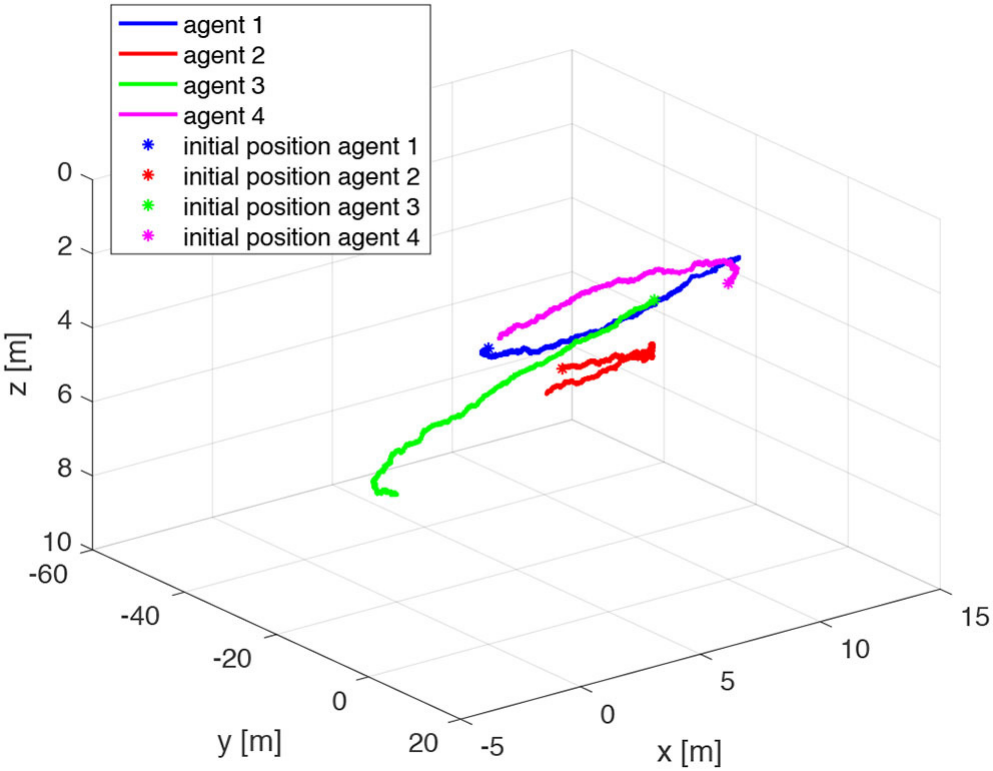
Trajectories of the agents for the simulation based on Relative Position Measurement Graph (RPMG) in Figure 13.

**Figure 15:**
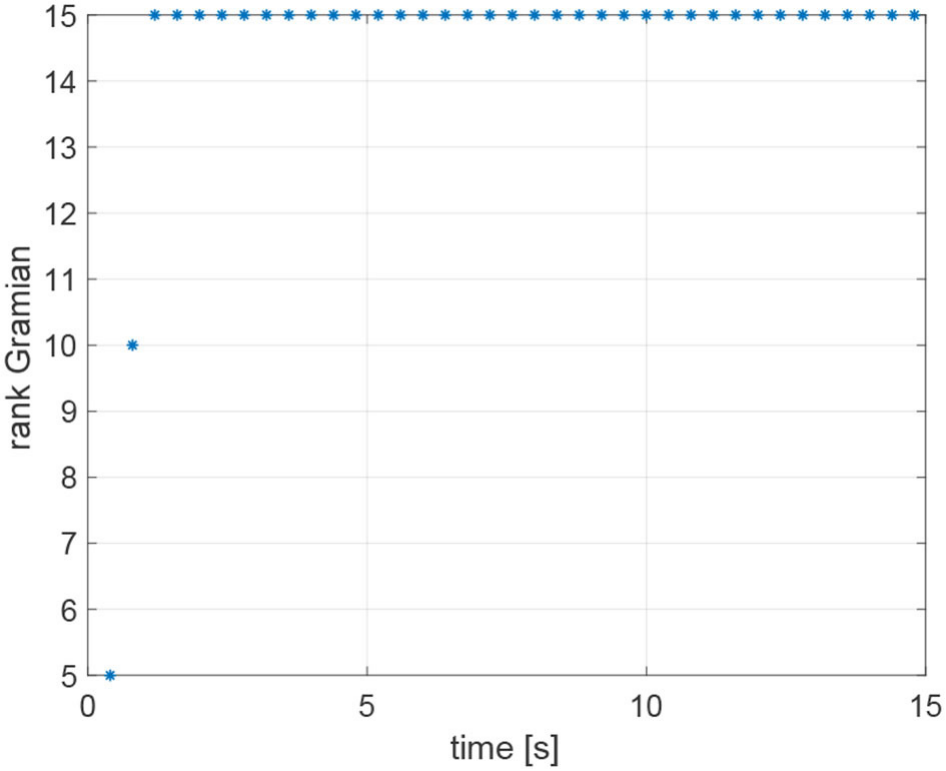
Rank of the observability Gramian for the simulation based on Relative Position Measurement Graph (RPMG) in Figure 13.

The initial positions of the agents are ${\text{x}}_{1}\left(0\right)={\left(0,0,2\right)}^{⊤}\text{m}$; ${\text{x}}_{2}\left(0\right)={\left(5,-10,4\right)}^{⊤}\text{m}$; ${\text{x}}_{3}\left(0\right)={\left(5,10,1\right)}^{⊤}\text{m}$; ${\text{x}}_{4}\left(0\right)={\left(10,0,2\right)}^{⊤}\text{m}$. The range measurements are assumed to be acquired with different time delays τ_*ij*_, namely τ_12_ = 0.1s, τ_24_ = 0.2s, τ_31_ = 0.3s, τ_23_ = 0.3s, τ_43_ = 0.4s, whereas a sampling time *T*_*s*_ = 0.4*s* has been considered. The resulting trajectories are shown in Figure 14. Notice that the observability Gramian (27) of the system is full rank along the trajectory of the vehicles, indeed rank(*G*) = 3*m* = 15 as shown in Figure 15. Therefore, given the observability of the system, the states **z**_*ij*_ can be estimated using the Kalman observer in Equations (32)–(36). The covariances of the state **z**_*ij*_ and the output ȳ employed in the Kalman filter are ***Q*** = 0.910^−5^·diag(1, 1, 1)m^2^ and *R* = 0.25m^2^, respectively. The initial Kalman filter state estimate is given by

(52) z^ij(0)~N(zij(0),Pij(0)), Pij(0)=9·diag(1,1,1)m2,

namely, **z**_*ij*_(0) is the initial true state and the initial condition z^ij(0) of the filter is assigned randomly with covariance ***P***_*ij*_(0).

**Figure 16:**
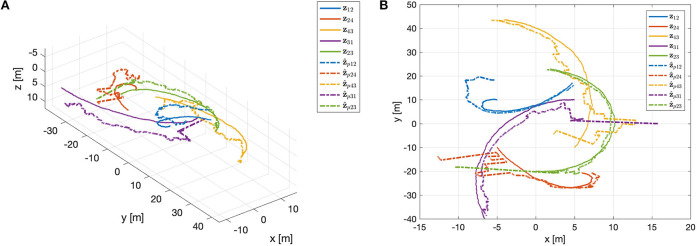
Constrained Kalman filter estimation of the relative motions: **(A)** 3D and **(B)** 2D.

Regardless of the specific approach adopted for the acoustic communications among agents, i.e., centralized or decentralized, the estimation of the relative positions ${\text{z}}^{*}={\left({\text{z}}_{12\;}^{⊤}{\text{z}}_{24\;}^{⊤}{\text{z}}_{43\;}^{⊤}{\text{z}}_{13\;}^{⊤};{\text{z}}_{23\;}^{⊤}\right)}^{⊤}\in {\mathbb{R}}^{15}$ is based on the Algorithm 1. Figure 16 reports the ultimate constrained Kalman filter estimate z^p* of the relative motions. It is worth remarking that, given the global observability of the motion, even if the estimations are initialized with a value far from the real one (see Equation 52), the resulting z^pij converge to **z**_*ij*_. This is an interesting feature because the proposed localization-oriented control law can actually be activated when the relative localization accuracy of agents is poor. Indeed, adopting such control strategy the whole agents network improves significantly its formation accuracy.

## 9. Conclusions

In this paper, the relative localization estimation problem for a team of vehicles is studied based on the knowledge of the range between agents of a subset of the participants. One main peculiarity of the proposed approach is that the relative velocity between agents, which is a fundamental data to solve the problem, is not assumed to be known in advance neither directly communicated. For this reason, a collaborative control protocol is designed in order to encapsulate the velocity data in the motion of each vehicle as a parameter through a dedicated control protocol, so that it can be inferred from the motion of the neighbor agents. Moreover, some suitable geometrical constraints associated with the agents' (unknown) positions are built and explicitly accounted for in the estimation schema providing a more accurate estimate. The issue of possible delays in the transmitted signals is also studied and two possible solutions are provided explaining how it is possible to get a reasonable range data exchange to get the solution both in a centralized fashion and in a decentralized one. Finally, the validity of the proposed approach is shown through numerical simulations.

## Data Availability Statement

The datasets generated for this study are available on request to the corresponding author.

## Author Contributions

All authors listed have made a substantial, direct and intellectual contribution to the work, and approved it for publication.

## Conflict of Interest

The authors declare that the research was conducted in the absence of any commercial or financial relationships that could be construed as a potential conflict of interest.
